# Rate of Infarct–Edema Growth on CT Predicts Need for Surgical Intervention and Clinical Outcome in Patients with Cerebellar Infarction

**DOI:** 10.1007/s12028-021-01414-x

**Published:** 2021-12-29

**Authors:** Yan Wang, Michael M. Binkley, Min Qiao, Amanda Pardon, Salah Keyrouz, Rajat Dhar, Andria L. Ford

**Affiliations:** Division of Stroke and Cerebrovascular Diseases, Departments of Neurology and Radiology, Washington University School of Medicine, Campus Box 8111, 660 S. Euclid Avenue, St. Louis, MO 63110-1093 USA

**Keywords:** Ischemic stroke, Cerebellar stroke, Brain edema, Risk

## Abstract

**Background:**

Up to 20% of patients with cerebellar infarcts will develop malignant edema and deteriorate clinically. Radiologic measures, such as initial infarct size, aid in identifying individuals at risk. Studies of anterior circulation stroke suggest that mapping early edema formation improves the ability to predict deterioration; however, the kinetics of edema in the posterior fossa have not been well characterized. We hypothesized that faster edema growth within the first hours after acute cerebellar stroke would be an indicator for individuals requiring surgical intervention and those with worse neurological outcomes.

**Methods:**

Consecutive patients admitted to the neurological intensive care unit with acute cerebellar infarction were retrospectively identified. Hypodense regions of infarct and associated edema, “infarct–edema”, were delineated by using ABC/2 for all computed tomography (CT) scans up to 14 days from last known well. To examine how rate of infarct–edema growth varied across clinical variables and surgical intervention status, nonlinear and linear mixed-effect models were performed over 2 weeks and 2 days, respectively. In patients with at least two CT scans, multivariable logistic regression examined clinical and radiological predictors of surgical intervention (defined as extraventricular drainage and/or posterior fossa decompression) and poor clinical outcome (discharge to skilled nursing facility, long-term acute care facility, hospice, or morgue).

**Results:**

Of 150 patients with acute cerebellar infarction, 38 (25%) received surgical intervention and 45 (30%) had poor clinical outcome. Age, admission National Institutes of Health Stroke Scale (NIHSS) score, and baseline infarct–edema volume did not differ, but bilateral/multiple vascular territory involvement was more frequent (87% vs. 50%, *p* < 0.001) in the surgical group than that in the medical intervention group. On 410 serial CTs, infarct–edema volume progressed rapidly over the first 2 days, followed by a subsequent plateau. Of 112 patients who presented within two days, infarct–edema growth rate was greater in the surgical group (20.1 ml/day vs. 8.01 ml/day, *p* = 0.002). Of 67 patients with at least two scans, after adjusting for baseline infarct–edema volume, vascular territory, and NIHSS, infarct–edema growth rate over the first 2 days (odds ratio 2.55; 95% confidence interval 1.40–4.65) was an independent, and the strongest, predictor of surgical intervention. Further, early infarct–edema growth rate predicted poor clinical outcome (odds ratio 2.20; 95% confidence interval 1.30–3.71), independent of baseline infarct–edema volume, brainstem infarct, and NIHSS.

**Conclusions:**

Early infarct–edema growth rate, measured via ABC/2, is a promising biomarker for identifying the need for surgical intervention in patients with acute cerebellar infarction. Additionally, it may be used to facilitate discussions regarding patient prognosis.

**Supplementary Information:**

The online version contains supplementary material available at 10.1007/s12028-021-01414-x.

## Introduction

The development of life-threatening edema occurs in up to 20% of patients with large cerebellar infarction [1, 2]. Given the limited space within the posterior fossa, malignant cerebellar infarction can result in secondary brainstem compression, obstructive hydrocephalus, coma, and eventually death, if left untreated. The long-term outcome after surgical decompression, however, can be favorable if treatment is performed in a time-sensitive manner [3–5].

Predicting which patients will deteriorate from cerebellar edema is challenging because of nonspecific symptoms and imaging pitfalls [6]. Early ischemic changes in the posterior fossa may be difficult to detect because of obscuration artifacts produced by bony structures of the skull base on computed tomography (CT). Admission clinical parameters such as age, National Institutes of Health Stroke Scale (NIHSS) score, and symptom-based triage, although helpful in anterior circulation strokes, have been shown to be unreliable predictors of deterioration in cerebellar infarcts [1, 3, 7–10]. Because of the low sensitivity of clinical characteristics, studies have recently investigated the use of advanced imaging, such as brain magnetic resonance imaging (MRI) and computed tomographic perfusion (CTP), to predict clinical decompensation. Quantitative baseline ischemic volume, measured on MRI or CTP, has been associated with clinical deterioration [8, 11]. Qualitative markers, such as hydrocephalus, brainstem deformation, and basal cistern compression, have also been described [12].

Much of the work mapping stroke edema formation and growth over time has only been explored in the anterior circulation. The absolute change in infarct–edema volume in the first 3–5 days after anterior circulation stroke has been associated with poor short-term and long-term outcomes [13]. Moreover, the rate of edema expansion has predicted clinical outcome [14, 15]. In contrast, observational data examining the kinetics of edema growth in patients with cerebellar infarcts are limited, preventing quantitative criteria to select patients who will benefit from escalation of care. Although CT images have limited pathological information, compared with MRI, serial CT scans are often readily available as part of standard of care. We postulated that quantification of evolving hypodense regions of infarct and associated edema, or “infarct–edema”, would increase diagnostic accuracy for malignant edema by informing how fast edema is developing. We hypothesized that, in addition to clinical variables and baseline infarct–edema volume, infarct–edema growth rate would predict (1) the need for surgical intervention and (2) worse clinical outcome in patients with cerebellar infarction.

## Methods

### Study population

Consecutive admissions to a neurological/neurosurgical intensive care unit (ICU) with acute cerebellar infarction from January 2006 through May 2019 were retrospectively identified. At our institution, the decision for ICU admission is made at the discretion of vascular neurologists and neurocritical care physicians. Patients are automatically admitted to the ICU if osmotic is delivered or if mechanical thrombectomy is performed within the past 24 h. Inclusion criteria for the study population were age ≥ 18 and ischemic stroke involving the cerebellum, confirmed by using CT imaging. Patients with isolated brainstem infarcts without visible cerebellar involvement on CT were not included in the initial database. Exclusion criteria included prior cerebellar stroke, concurrent cerebral ischemic stroke greater than 50% of the MCA territory, symptomatic hemorrhagic transformation (parenchymal hematoma type 1 or parenchymal hematoma type 2, based on the European Cooperative Acute Stroke Study II classification) [16], and decision to pursue comfort care within 7 days of admission without receiving maximal medical or surgical therapy. The study was approved by the local institutional review board, with a waiver of written informed consent.

### Data Collection and Clinical End Points

Clinical and radiological variables were abstracted retrospectively from medical records. Clinical and demographic information included age, sex, time of last known well (LKW), thrombolytic treatment, NIHSS score and Glasgow Coma Scale (GCS) score at admission to ICU, admission lab values (glucose, creatinine), chart documented medical history (prior ischemic stroke, hypertension, hyperlipidemia, diabetes mellitus, coronary artery disease, and atrial fibrillation/flutter), time of medical and/or surgical intervention, discharge NIHSS score, discharge GCS score, and discharge location. For patients who were deceased at the time of discharge, an NIHSS of 42 and GCS of 3 were used.

Radiological data extracted from CT imaging included time of acquisition, vascular territory involvement, presence of brainstem infarct, and cerebellar infarct–edema volume. For simplicity, infarct territory classification was collapsed into binary categories: infarcts involving either bilateral or multiple cerebellar vascular territories versus infarcts involving one vascular territory [17]. We also used clinical MRI, if available, to define brainstem infarction, given the limited resolution on CT. All CT scans from admission up to time of final intervention therapy (osmotic, extraventricular drainage [EVD], or suboccipital decompressive craniectomy [SDC]) or to day 14 from LKW, whichever was the earliest, were included.

The treatment end point was defined by intervention type. Patients who remained stable on conservative medical management with or without osmotic (mannitol, 5% sodium chloride, or 23.4% sodium chloride) therapy were included in the “medical group”. Patients who underwent surgical interventions (EVD and/or SDC), in addition to medical management, were included in the “surgical group”. In our institutional practice, osmotic therapy is not used prophylactically, but it is initiated at the time of level of consciousness worsening in association with edema with mass effect on CT scan. Patients who had a decline in neurological status, despite maximal medical therapy, underwent surgical decompression based on previously published guidelines [1, 18].

The clinical outcome end point was defined by hospital discharge location. “Good outcome” was defined as discharge to acute inpatient rehabilitation hospital or discharge to home, whereas “poor outcome” was defined as discharge to a skilled nursing facility, long-term acute care facility, hospice, or morgue. Although 30-day and 90-day modified Rankin Scale scores were not uniformly available because of the retrospective nature of our clinical database, discharge destination has been used as a reliable surrogate for modified Rankin Scale in stroke survivors [19].

### Image Analysis for Infarct–Edema Volume

Imaging interpreters were blinded to surgical and clinical outcomes. Infarct-related hypodensity volume was manually measured on clinical CT scans. Visible regions of infarct-related hypodensity in the cerebellum likely represented a combination of infarct core and edematous tissue. This region is referred to as “infarct–edema” throughout the article, with the understanding that the delineated lesion may include both injured brain tissue and reactive fluid components. The ABC/2 formula was used to measure volume, where A is the longest lesion axis of any image slice, B is the line perpendicular to A at the widest dimension, and C is the height, obtained by multiplying number of CT slices by individual slice thickness. In cases of bilateral cerebellar hemispheric infarcts, individual hypodensity volumes were measured separately and added. It provides the best simple geometric estimate of hemorrhagic stroke volume and infarct volume [20, 21].

First, to validate the ABC/2 formula as a representative estimate of infarct–edema volume in our study population, two neurologists (Y.W. and A.P.) measured infarct–edema volume on 15% of sample CTs by using the ABC/2 method and a volumetric method. The volumetric method was defined as volume (ml) = (A1 + A2 + A3 + ··· + A*n*) × H, where *n* indicates the number of image sections showing region of interest, A indicates the area (cm^2^), manually delineated for each image slice, and H indicates the height (cm) defined by slice thickness. The concordance between the ABC/2 method and the volumetric method was assessed by using Lin’s concordance coefficient (ρ_c_), revealing a good concordance of 0.87 (95% confidence interval [CI] 0.80–0.92).

Second, to ensure the eventual prediction model could be readily applied in acute care setting, two neurologists (Y.W. and M.Q.) used the more pragmatic ABC/2 method to measure infarct–edema volume for all remaining CTs. A randomly selected 9% sample of CT scans yielded an intraclass correlation of 0.92 (95% CI 0.86–0.96), indicating excellent interrater reliability.

### Statistical Analysis

Descriptive statistics of baseline variables were reported as percentages for categorical data and as medians with interquartile ranges for continuous data. Statistical analyses for group comparisons and univariate analysis were performed using the χ^2^ test or Fisher’s exact test and Mann–Whitney *U*-test for categorical and continuous variables, respectively. Using the univariate results, two multivariable logistic regression models were constructed to determine baseline clinical and radiological predictors of surgical intervention, with forward stepwise entry for variables with *p* value < 0.3, and *p* value < 0.05 to be retained. Logistic regression collinearity and final model validity were checked. Collinearity between variables was assessed by using the variance inflation factor. None of our variables exhibited a variance inflation factor > 10 or |r|> 0.6, which would have required exclusion from the model. Fit of logistic regression models was assessed by using the Hosmer–Lemeshow test. The variance explained by the model was calculated using R^2^. Statistical analyses were performed using SAS software version 9.4 (SAS Institute Inc, Cary, NC).

### Modeling Infarct–Edema Growth Trajectory

To model the trajectory of infarct–edema growth, infarct–edema volume as a function of time from LKW was plotted as a spaghetti plot for the 410 head CTs over the 14-day period. Differences in rate of infarct–edema growth between individuals who received conservative medical management and individuals who required treatment escalation and underwent surgical intervention were compared by using two models: (1) a nonlinear (logistic curve) model to capture the steep rise and long plateau over 2 weeks and (2) a linear mixed-effects model to fit the linear rate of growth over the first 2 days. The former nonlinear mixed-effect model allowed for estimation of infarct–edema growth rates and asymptotic (plateau) volumes of patients who received medical management vs. patients who received surgical intervention while accounting for repeated measures [22, 23]. It also accounted for the variability in time to ED presentation among patients. The spaghetti plot of infarct–edema volume over time demonstrated a greater rate of change (steeper slope) in the first 48 h. Therefore, we then performed the second of the two models by using a linear mixed-effect model to describe this early critical period within the first 2 days, while excluding data from the later, more stable period. The estimated cut point, in which the growth rate transitioned from linear to constant, was found to be 2.1 days, by using a single knot, monotonic spline fit. This model also adjusted for variability in time to ED presentation.

### Prediction of Surgical Intervention and Poor Clinical Outcome

Lastly, to determine whether infarct–edema growth rate predicted surgical intervention or poor clinical outcome (independent of baseline infarct–edema volume and other clinical covariates), we first calculated growth rate as the change in infarct–edema volume over the change in time. We used a subgroup of patients who had at least two scans within the first 2 days. In patients with three or more CT scans, the two scans performed closest to LKW and closest to 48 h from LKW were selected. On sensitivity analysis, there were no differences in demographics, admission NIHSS, past medical history, and surgical intervention frequency between participants who were included in and those who were excluded from the subanalysis (data not shown). We used two multivariable logistic regression models to determine whether early infarct–edema growth rate was predictive of surgical intervention and clinical outcome, independent of baseline infarct–edema volume. We used forward stepwise entry for variables with a *p* value < 0.3 and a *p* value < 0.05 to be retained. The logistic regression analyses were then adjusted for covariates (baseline infarct–edema volume, bilateral or multiple vascular territories, and baseline NIHSS). Additionally, we adjusted for concurrent brainstem infarct as a covariate in clinical outcome. Receiver operating characteristic analysis was performed with and without infarct–edema growth rate and area under the curve was calculated.

## Results

### Clinical and Radiological Imaging Characteristics of Patients with Acute Cerebellar Infarction

**Fig. 1:**
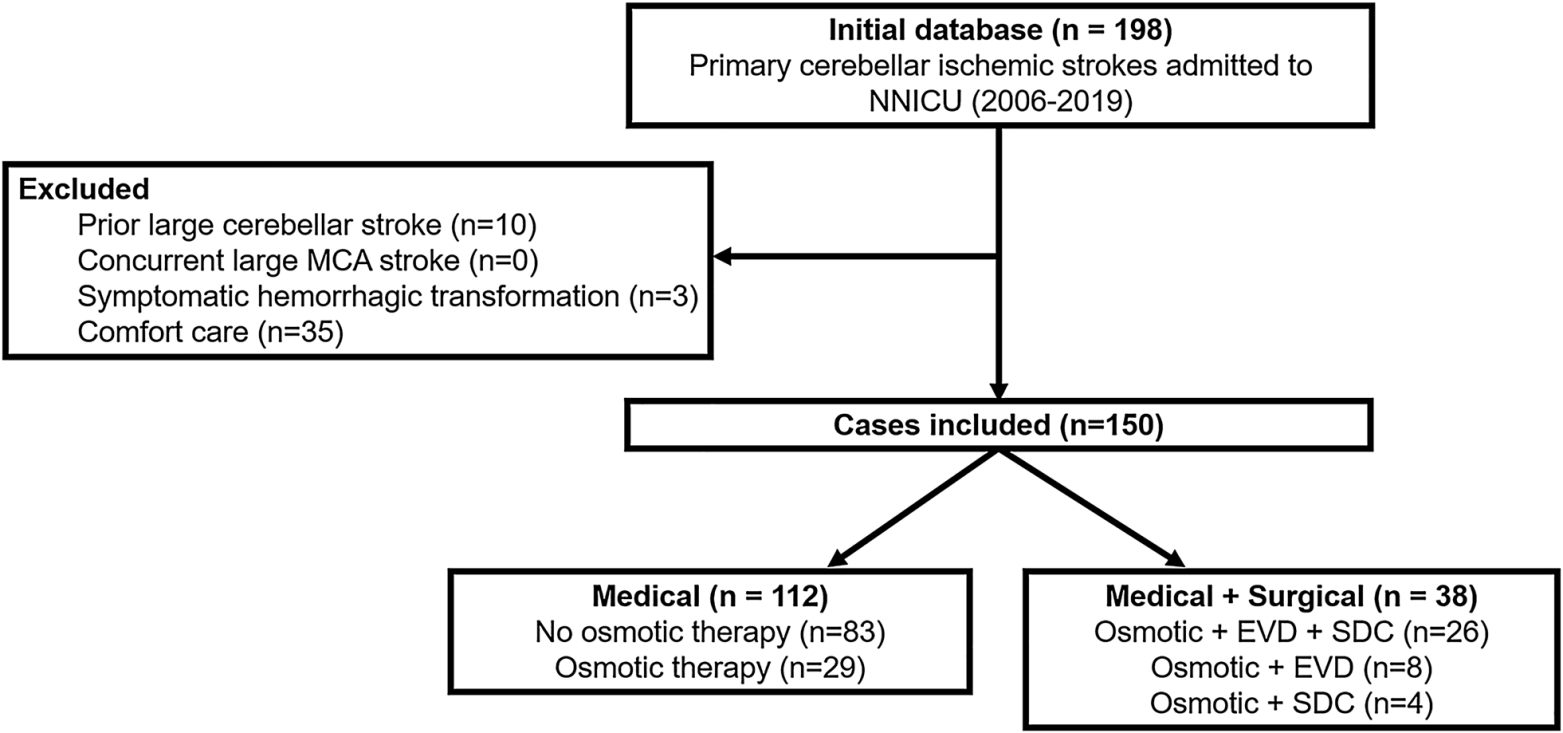
Participant enrollment flow chart and reasons for study exclusion. Patients with acute cerebellar ischemic stroke admitted to the neurological ICU from 2006 to 2019 were considered for inclusion. CT, computed tomography, EVD, external ventricular drain, ICU, intensive care unit, NNICU, neurology and neurosurgery ICU, SDC, suboccipital decompressive craniectomy

From 2006 to 2019, 198 patients were admitted to the neurological ICU with primary cerebellar infarction confirmed at imaging. Of these, 150 met final eligibility criteria (reasons for exclusion are shown in Fig. 1). One hundred and twelve (75%) patients underwent medical management, of which 29 patients received osmotic therapy in addition to ICU monitoring. Of the 38 patients who received surgical intervention, 30 ultimately underwent SDC (Fig. 1). Baseline characteristics and clinical outcomes for the medical and surgical intervention groups are shown in Table 1. Most patients in both groups presented to the ED beyond the thrombolytic time window for ischemic stroke (medical 1.02 [0.30, 1.98] day vs. surgical 0.83 [0.14, 2.41] day, *p* = 0.65). As a result, only 9.8% and 5.3% of patients received a tissue plasminogen activator in the medical and surgical groups, respectively (*p* = 0.52). One hundred twenty-five patients were admitted directly to the ICU from the ED, whereas 25 patients were transferred from an inpatient ward to the ICU after clinical decline. Only one patient had an EVD inserted prior to the transfer. On admission to the ICU, compared with the medical group, the surgical group had (nonsignificantly) more severe neurological impairment measured by GCS (*p* = 0.06) and by NIHSS (*p* = 0.09). In the surgical group, EVD and SDC occurred on day 2.5 (1.5, 4.6) and 2.6 (1.5, 5.2), respectively.

**Table 1:** Clinical and radiographic features between medical and surgical cohort

Characteristics	Medical (*n* = 112)	Surgical (*n* = 38)	*p* value
Age (year)	60 (52–73)	60 (53–69)	0.57
Female sex	43 (38)	14 (37)	1.00
African American	45 (42)	11 (32)	0.40
Prior comorbidities			
Prior stroke	24 (22)	8 (22)	1.00
Hypertension	88 (79)	28 (74)	0.51
Coronary artery disease	33 (30)	14 (37)	0.43
Atrial fibrillation/flutter	15 (14)	8 (22)	0.30
Diabetes mellitus	48 (43)	17 (45)	0.85
Hyperlipidemia	43 (39)	18 (49)	0.34
Imaging findings			
Baseline infarct–edema volume (ml)	11.4 (0–22.5)	25.9 (0–34.2)	0.17
Brainstem infarct	39 (35)	9 (24)	0.23
Hemorrhagic transformation^a^	12 (11)	9 (24)	0.06
Infarct vascular territory			0.001*
Bilateral or multiple	56 (50)	33 (87)	< 0.0001*
PICA only	39 (35)	5 (13)	0.01*
AICA only	2 (2)	0 (0)	1.00
SCA only	15 (13)	0 (0)	0.02*
Hospital course			
Time to ED presentation	1.02 (0.30–1.98)	0.83 (0.14–2.41)	0.65
tPA	11 (9.8)	2 (5.3)	0.52
Mechanical thrombectomy	9 (8.0)	4 (10.5)	0.74
GCS	14 (10.5–15)	13.5 (7–15)	0.06
NIHSS	5.5 (2–18)	8 (4–23)	0.09
Glucose (mg/dl)	154 (118–226)	160 (130–197)	0.75
Creatinine (mg/dl)	1.0 (0.8–1.3)	1.0 (0.8–1.1)	0.39
Time to osmotic (days)	3.2 (1.2–4.5)	2.2 (1.4–4.6)	0.84
Time to EVD (days)	–	2.5 (1.5–4.6)	–
Time to SDC (days)	–	2.6 (1.5–5.2)	–
Clinical outcome			
Δ GCS	0 (0–1)	0 (−1–1.25)	0.86
Δ NIHSS^c^	−1 (-3–1)	−1 (−7–5.5)	0.81
Discharge location			0.15
Inpatient rehabilitation	64 (57)	21 (55)	0.85
Home	18 (16)	2 (5)	0.11
Skilled nursing facility	7 (6)	6 (16)	0.09
Long-term acute care	6 (5)	4 (11)	0.27
Hospice or morgue	17 (15)	5 (13)	1.00

Data presented are number (%) for categorical and median (IQR) for continuous variables

“Delta NIHSS and delta GCS” reflect changes in neurological scales from admission to the ICU to hospital discharge. For patients who were deceased at the time of discharge, a NIHSS of 42 and GCS of 3 were used

AICA, anterior inferior cerebellar artery, ED, emergency department, EVD, extraventricular drainage, GCS, Glasgow Coma Scale, IQR, interquartile range, NIHSS, National Institutes of Health Stroke Scale, PICA, posterior inferior cerebellar artery, SCA, superior cerebellar artery, tPA, tissue plasminogen activator

^*^Indicates a significant *p* value

^a^Asymptomatic, hemorrhagic infarction type 1 or hemorrhagic infarction type 2 based on the European Cooperative Acute Stroke Study II classification

^b^Time from last known well

^c^Discharge NIHSS values were missing in seven medical cohort participants

Baseline infarct–edema volume, measured by using the ABC/2 method, was delineated on the first CT performed at the time of initial stroke evaluation. Because of the difficulty of detecting early ischemic changes on CT, infarct-related hypodensity was visualized on 84 (75%) and 26 (68%) of the initial CTs in the medical and surgical groups, respectively (*p* = 0.43). As a result, baseline infarct–edema volume was not different between the two groups (medical 11.4 [0, 22.5] ml vs. surgical 25.9 [0, 34.2] ml, *p* = 0.17). However, in the group of patients with visible infarct–edema on initial CT, the surgical group had greater baseline infarct–edema volume (medical 18.4 [8.8, 25.2] ml vs. surgical 33.7 [18.8, 46.8] ml, *p* = 0.001). Patients in the surgical group were more likely to have infarct–edema involving bilateral or multiple vascular territories that those in the medical group (*p* < 0.0001; Table 1).

At the time of discharge, more than half of patients from both intervention groups were discharged to inpatient rehabilitation facilities (medical 57% vs. surgical 55%, *p* = 0.85), whereas more patients from the surgical group were discharged to skilled nursing facilities, trending significance (*p* = 0.09).

### Radiographic Features, But not Baseline Clinical Features, Predicted Patients Who Received Surgical Intervention

Prior to examining infarct–edema growth, we performed univariate and multivariable logistic regression to identify baseline predictors of patients who received surgical intervention. On univariate analysis, of all the clinical and radiological predictors, only baseline infarct–edema volume (OR 1.32; 95% CI 1.07–1.64), bilateral or multiple vascular territories (OR 6.60; 95% CI 2.40–18.1), and hemorrhagic transformation on admission CT (OR 2.59; 95% CI 0.99–6.74) were associated with surgical intervention. On multivariable logistic regression analysis, baseline infarct–edema volume and bilateral or multiple vascular territories remained independent predictors of surgical intervention (Table 2). This predictive model explained 20% of the variance in surgical intervention. Patient demographics, past medical history, and initial NIHSS/GCS did not predict surgical intervention.

**Table 2:** Univariate and multivariable analysis of baseline clinical and radiological predictors of patients receiving surgical intervention for acute cerebellar infarction

Variable	Univariate		Multivariable	
	OR (95% CI)	*p* value	OR (95% CI)	*p* value
Baseline infarct–edema volume (per 10-ml increase)	1.32 (1.07–1.64)	0.01*	1.26 (1.01–1.58)	0.039*
Bilateral or multiple vascular territories	6.60 (2.40–18.1)	< 0.0001*	5.65 (1.93–16.50)	0.002*
Hemorrhagic transformation	2.59 (0.99–6.74)	0.047*	–	–
NIHSS	1.02 (0.99–1.05)	0.18	1.00 (0.97–1.04)	0.91
GCS	0.94 (0.85–1.03)	0.17	–	–

CI, confidence interval, GCS, Glasgow Coma Scale, NIHSS, National Institutes of Health Stroke Scale, OR, odds ratio.

*Indicates a significant *p* value.

### Infarct–Edema Progressed Rapidly in the First 2 Days, with Subsequent Plateau Over 2 Weeks

**Fig. 2:**
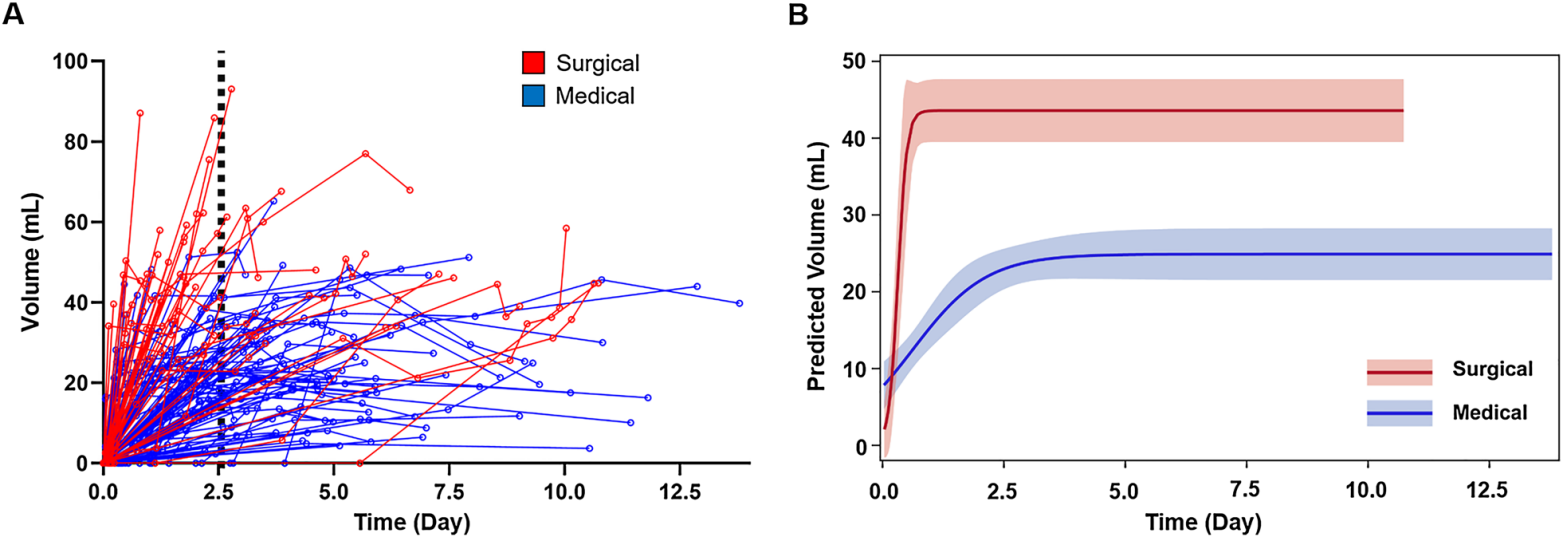
Temporal evolution of infarct–edema volume by intervention status. Infarct–edema volumes were measured on 410 clinical CT scans over 14 days from last known well (LKW). **a** Spaghetti plot of infarct–edema volume as a function of LKW by intervention status. EVD and SDC occurred a median 2.5 and 2.6 days from LKW, respectively (black dotted line). **b** A nonlinear mixed model was used to best describe the differences in infarct–edema growth pattern between the medical and surgical groups. For both groups, infarct–edema expanded rapidly early on, with subsequent plateau in volume. The surgical group reached a higher predicted plateau volume than that of the medical group (43.6 ml [95% CI 38.3–48.8] vs. 24.9 ml [95% CI 21.5–28.3], *p* < 0.0001). Logistic nonlinear mixed model regression and 95% CIs are shown per group. CI, confidence interval, CT, computed tomography, EVD, external ventricular drain, SDC, suboccipital decompressive craniectomy

Because of the low variance explained by baseline infarct–edema volume and infarct territory involvement at predicting patients requiring surgical intervention, we examined the added value of infarct–edema growth rate, as measured on serial CTs. First, to describe the temporal evolution of infarct–edema over the first 14 days, we plotted infarct–edema volume as a function of time from LKW across all 410 scans in 150 patients (Fig. 2a, one line per patient). Next, we modeled infarct–edema growth of the medical and surgical groups (Fig. 2b). In both groups, infarct–edema volume was characterized by initial rapid growth followed by subsequent plateau. Peak infarct–edema growth rate was faster in those destined for surgical intervention compared with the medical group (*p* = 0.005). The final predicted mean plateau volume was 24.9 ml (95% CI 21.5–28.3) and 43.6 ml (95% CI 38.3–48.8) for the medical and surgical groups, respectively (*p* < 0.0001).

### Infarct–Edema Growth was Faster in Patients Receiving Surgical Intervention

**Fig. 3:**
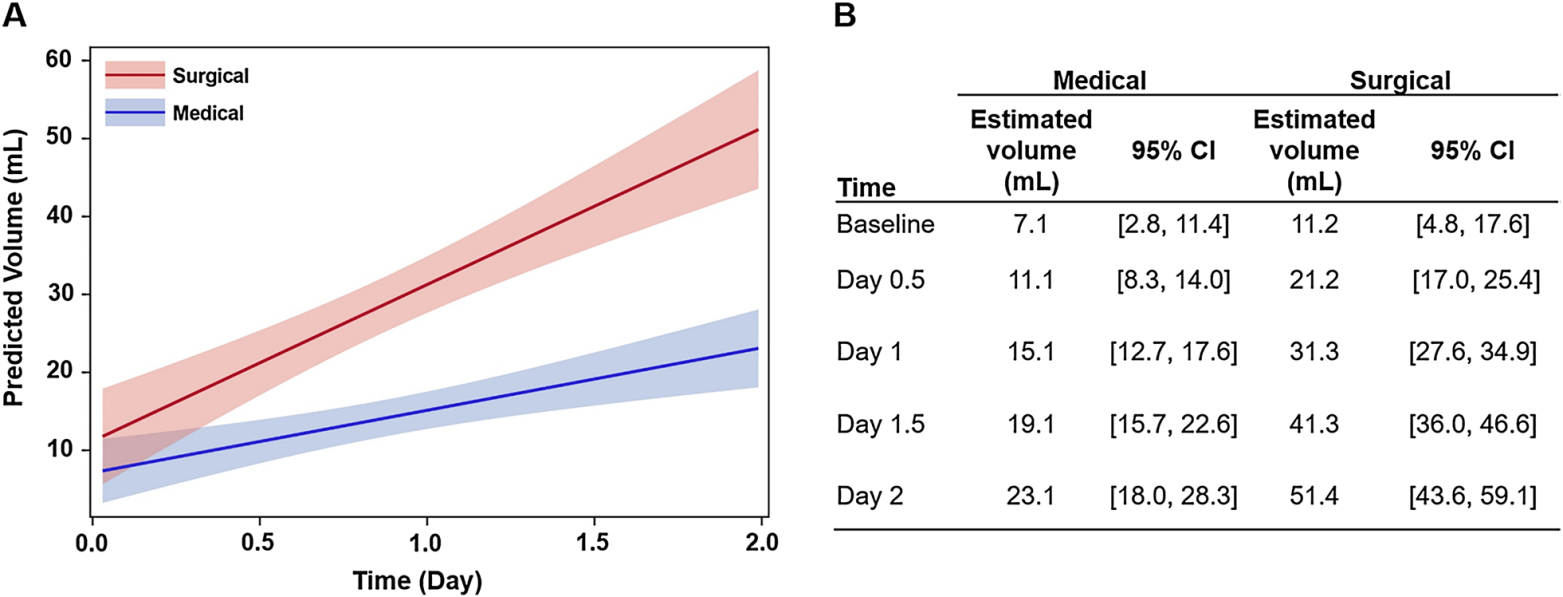
Rate of infarct–edema growth was greater in the patients who received surgical interventions. **a** One hundred eighty-three CT scans from 112 patients were performed within 2 days of LKW. Early volumetric change for the medical and medical groups was best described by a linear mixed model. Compared with medical group, infarct–edema growth rate was significantly faster in the surgical group (medical group, 8.01 ml/day [95% CI 0.7–15.3]; surgical group, 20.1 ml/day [95% CI 14.0–26.2], *p* = 0.002). A significant interaction between intervention and time was observed (time intervention *p* = 0.002). Effect plot of time versus predicted infarct–edema volume is shown with 95% CIs. **b** Estimated infarct–edema volumes of the medical and surgical groups at different time points since LKW were shown. The surgical group exhibited greater infarct–edema volume starting on Day 0.5. CI, confidence interval, CT, computed tomography, LKW, last known well

Although infarct–edema growth followed a logistic growth pattern over the 2-week period, we observed a steep linear growth pattern over the first 2 days. Thus, we further characterized the infarct–edema growth pattern during this early criterial period. One hundred twelve patients had at least one CT scan (183 scans total) over the first 2 days of LKW. Compared with the medical group, the surgical group was associated with greater infarct–edema volume by day 0.5 and onward (Fig. 3b). Infarct–edema growth rate was significantly faster in the surgical group than that in the medical group (medical group 8.01 ml/day [95% CI 0.7–15.3]; surgical group: 20.1 ml/day [95% CI 14.0–26.2], *p* = 0.002, Fig. 3a). The interaction remained significant after adjusting for age (data not shown).

### Infarct–Edema Growth Rate Remained an Independent and the Strongest Predictor of Surgical Intervention

Next, we evaluated if the addition of early infarct–edema growth rate improved the ability to predict individuals requiring surgical intervention. We first calculated the rate of growth on the subset of patients (*n* = 67) who had at least two CT scans (134 scans total) in the first 2 days since LKW (Supplemental Table 1). The calculated infarct–edema growth rates of the medical and surgical groups were consistent with the growth rates modeled in Fig. 3. On multivariable logistic regression, the odds of undergoing surgical intervention increased by 2.55 for every 10 ml/day increase in infarct–edema growth rate within the first 48 h, adjusting for baseline infarct–edema volume, bilateral or multiple vascular territories, and admission NIHSS (Table 3). The addition of infarct–edema growth rate to the multivariable model containing baseline infarct–edema volume, vascular territories, and NIHSS, improved the area under the curve from 0.68 (95% CI 0.54–0.81; *p* = 0.02) to 0.91 (95% CI 0.82–0.99; *p* < 0.001) (*p* = 0.0002). The final multivariable model explained 49% of the variance in the intervention end point, relative to 20% variance explained in the previous model including only baseline variables.

**Table 3:** Univariate and multivariable analyses of clinical and radiological predictors of surgical intervention in patients with at least two CTs within 2 days of LKW

Variable	Univariate		Multivariable	
	OR (95% CI)	*p* value	OR (95% CI)	*p* value
Infarct–edema growth rate (per 10 ml/day)	1.60 (1.16–2.21)	0.005*	2.55 (1.40–4.65)	0.002*
Baseline infarct–edema volume (per 10-ml increase)	1.14 (0.77–1.69)	0.509	2.34 (1.18–4.64)	0.015*
Bilateral or multiple cerebellar vascular territories	8.75 (1.83–41.94)	0.007*	9.62 (1.50–61.77)	0.017*
Hemorrhagic transformation	1.78 (0.43–7.41)	0.430	–	–
NIHSS	1.03 (0.99–1.08)	0.191	0.98 (0.91–1.04)	0.49
GCS	0.91 (0.81–1.04)	0.156	–	–

CI, confidence interval, CT, computed tomography, GCS, Glasgow Coma Scale, LKW, last known well, NIHSS, National Institutes of Health Stroke Scale, OR, odds ratio.

*Indicates a significant *p* value.

### Rapid Infarct–Edema Growth was Independently Associated with Poor Clinical Outcome

We explored the clinical and radiological predictors of poor clinical outcome within our study population and evaluated if early infarct–edema growth rate was associated with worse clinical outcome. In the subgroup of patients with at least two CTs over the first 2 days, clinical and radiological variables with poor versus good clinical discharge outcomes are shown in Supplemental Table 2. On univariate logistic regression analysis, infarct–edema growth rate (OR 2.09; 95% CI 1.39–3.16), admission NIHSS (OR 1.12; 95% CI 1.05–1.18), and brainstem infarct (OR 3.90; 95% CI 1.34–11.37) were associated with poor outcome, whereas baseline infarct–edema volume and bilateral or multiple vascular territories were not. On multivariable logistic regression, infarct–edema growth rate remained a significant predictor of poor outcome, independent of baseline infarct–edema volume, NIHSS, brainstem infarct, and vascular territories (Table 4). The odds of discharge to unfavorable locations (SNF, LTAC, hospice, or morgue) increased by a factor of 2.2 for every 10 ml/day increase in infarct–edema growth rate within 48 h.

**Table 4:** Univariate and multivariable analyses of clinical and radiological predictors of poor outcome, patient subgroup with at least two CTs within 2 days of LKW

Variable	Univariate		Multivariable	
	OR (95% CI)	*p* value	OR (95% CI)	*p* value
Infarct–edema growth rate (per 10 ml/day)	2.09 (1.39–3.16)	< 0.001*	2.20 (1.30–3.71)	0.003*
Baseline infarct–edema volume (per 10 ml)	0.70 (0.44–1.10)	0.12	1.20 (0.60–2.42)	0.60
Brainstem infarct	3.90 (1.34–11.37)	0.01*	3.56 (0.62–20.25)	0.15
Bilateral or multiple vascular territories	1.96 (0.65–5.94)	0.23	0.32 (0.06–1.81)	0.20
NIHSS	1.12 (1.05–1.18)	< 0.001*	1.10 (1.02–1.18)	0.019*
Surgical intervention	1.85 (0.65–5.27)	0.25	–	–
Age	0.98 (0.95–1.02)	0.33	–	–
Mechanical thrombectomy	4.44 (0.99–19.79)	0.05	–	–
Prior stroke	2.63 (0.85–8.11)	0.09	–	–

CI, confidence interval, CT, computed tomography, LKW, last known well, NIHSS, National Institutes of Health Stroke Scale, OR, odds ratio.

*Indicates a significant *p* value.

## Discussion

We found that imaging metrics, rather than baseline clinical metrics, were stronger predictors of surgical intervention in patients with large cerebellar strokes. Baseline infarct–edema volume and vascular territory, however, explained only 20% of the variance in treatment escalation. As serial noncontrast head CTs are routinely acquired and readily available, we investigated the kinetics and added diagnostic value of early edema formation following cerebellar infarction. Within the first 2 days, patients who eventually underwent surgical intervention exhibited 2.5 times faster infarct–edema volume growth rate than patients who remained medically managed. Further, the likelihood of receiving surgical intervention more than doubled for every 10 ml/day increase in infarct–edema growth rate. The addition of infarct–edema growth rate to the predictive model for intervention type improved the variance explained from 20 to 49%. Additionally, the risk of discharge to unfavorable locations doubled for every 10 ml/day increase in infarct–edema growth rate within 48 h, independent of baseline infarct–edema volume, admission NIHSS, and brainstem infarct. Thus, improving our understanding of malignant edema growth may help guide optimal management in acute cerebellar infarction.

Our results have several practical implications. First, our findings are consistent with and reaffirm current recommendations on the importance of performing serial CTs within the first two days to identify patients at high risk of developing malignant edema [1]. Based on the mean and 95% CI of infarct–edema growth rates in Fig. 3a, patients with a growth rate greater than 14–20 ml/day (or 7–10 ml per 12 h) require closer monitoring and may benefit from early neurosurgical consultation. Second, our data support the practice of using ABC/2 equation to determine infarct–edema size during acute care settings [21]. The equation allows for rapid, bedside quantification of infarct–edema volume and growth rate, which in turn can be used to predict surgical intervention needs and clinical outcome at discharge. If these results are confirmed in prospective studies, infarct–edema growth rate may be considered for incorporation into guidelines regarding the optimal management of patients with malignant cerebellar stroke.

Of the radiological variables, rate of infarct–edema growth, baseline infarct–edema volume and bilateral or multiple vascular territories predicted patients requiring surgical intervention. We demonstrated greater infarct–edema volume and faster infarct–edema growth rate in the surgical group as early as day 0.5, with the majority of infarct–edema growth occurring within the first 2 days. Our baseline infarct–edema volume results were consistent with previously reported values. In patients who later developed malignant cerebellar edema, baseline infarct volume ranged from 22 to 33 ml, involving 40% of one cerebellar hemisphere or multiple vascular territories [8, 11, 12]. Importantly, we found that infarct–edema growth rate was a stronger early predictor of surgical intervention than baseline infarct–edema volume and vascular territory involvement. The addition of infarct–edema growth rate to the latter two radiological variables significantly improved the differentiation of patients who did and did not receive surgical interventions.

Consistent with prior research [3, 8, 11], we did not find clinical variables, such as age, NIHSS, and medical comorbidities, as significant predictors of surgical intervention in patients with cerebellar stroke. In anterior circulation strokes, younger age and higher NIHSS on admission have been associated with early edema formation and clinical decompensation [24]. Such contrasting results are expected, as NIHSS encapsulates mainly anterior circulation stroke symptoms and posterior circulation stroke symptoms are often nonspecific. Our study highlights the poor reliability of clinical characteristics as predictors of malignant cerebellar edema requiring surgical intervention. Additional research is warranted to focus specifically on posterior circulation strokes, as conclusions drawn from the anterior circulation stroke literature may not be applicable to the posterior circulation.

In addition to being an early predictor of surgical intervention, faster infarct–edema growth rate was also associated with poor clinical outcome, independent of baseline infarct–edema volume. This suggests that subsequent cytotoxic edema may play a larger role in determining clinical outcome than initial ischemic insult alone. This hypothesis was supported by previous literature on moderate-sized anterior circulation ischemic stroke, where absolute change in edema volume, independent of baseline infarct volume on diffusion weighted imaging, predicted poor 90-day outcome [13]. Indeed, a recent clinical trial focusing on reducing cerebral edema growth pharmacologically in large hemispheric strokes was associated with favorable radiological and clinical end points [25].

Our study has several advantages. Compared with previous studies using advanced imaging techniques when evaluating infarct volume [8, 11, 12], our design was pragmatic and robust in its use of 410 noncontrast CT scans. Although MRI and CTP are more sensitive than conventional CT at detecting posterior fossa strokes at earlier time points, advanced imaging techniques are less likely to be performed as readily across clinical settings and are challenging to obtain serially when a patient is neurologically unstable. Our study has several limitations. Clinical and imaging data availability were limited by the study’s retrospective, single center study design. Conclusions drawn from our study were influenced by institutional practice. A multicenter study is needed to validate the clinical utility of infarct–edema growth rate within a larger, independent sample of patients. Additionally, although the CT protocol and imaging database used in our study were not prespecified and standardized, the inclusion of CT images from a variety of sources allows for generalizability. Our baseline infarct–edema volume variable may be confounded as a result of difficulty detecting early ischemic changes on CT. To control for this confounder, we examined a subgroup of patients with infarct–edema volume visible on initial CT. On multivariable analysis, baseline infarct–edema volume remained a significant predictor of surgical intervention after adjusting for bilateral or multiple vascular territories and NIHSS (data not shown). Furthermore, we did not incorporate serial neurological examinations into our predictor model for surgical intervention. Although serial NIHSS may not be a strong clinical predictor given the scale captures mostly anterior circulation symptoms, serial examinations targeting specific brainstem signs and level of consciousness may provide additional predictive value. Lastly, we used hospital discharge location as surrogate marker of clinical outcome as we did not have access to post-hospital discharge modified Rankin scale scores. Prior studies have shown discharge destination was a suitable **s**urrogate for 3-month and 12-month Modified Rankin Scale scores in poststroke patients [19].

## Conclusions

The infarct–edema growth rate over the first 48 h independently predicted the need for surgical intervention in patients with cerebellar infarction. Understanding the kinetics of cerebellar edema and its contribution to patient decompensation will allow clinicians to identify patients at the earliest opportunity, allowing for informed decision making in the management of malignant edema and facilitating family discussions.

## Supplementary Information

Below is the link to the electronic supplementary material.

Supplementary file1 (DOCX 29 kb)

Supplementary file2 (DOCX 95 kb)

## Data Availability

Data are available on request to the corresponding author.
