# Supplementary material for: Rate of Infarct–Edema Growth on CT Predicts Need for Surgical Intervention and Clinical Outcome in Patients with Cerebellar Infarction
Source: Neurocrit Care. 2021 Dec 29;36(3):1011–21. doi: 10.1007/s12028-021-01414-x (PMC9110544; doi:10.1007/s12028-021-01414-x)
Supplement: Supplementary file 1 — Supplementary file1 (DOCX 29 kb) [file 12028_2021_1414_MOESM1_ESM.docx]

| **Supplemental Table 1. Imaging characteristics of patients with at least 2 CT within 2 days of LKW** | | | | | |
| --- | --- | --- | --- | --- | --- |
| Characteristics | Medical  (n = 45)  Median [IQR] | Surgical  (n = 22)  Median [IQR] | *P* value | |  |
| CT_baseline_ infarct-edema volume, mL​ | 6.7 [0, 18.6] | 2.8 [0, 26.8] | 0.89 | |  |
| CT_endpoint_ infarct-edema volume, mL | 21.9 [14.3, 25.1] | 44.2 [34.8, 50.5] | **<0.0001** | |  |
| Time to CT_baseline_, day^a^ | 0.42 [0.24, 1.03] | 0.16 [0.10, 0.53] | **0.02** | |  |
| Time to CT_endpoint_, day^a^ | 1.69 [1.22, 2.07] | 1.51 [1.13,1.84] | 0.26 | |  |
| Δ Time, day | 0.95 [0.47, 1.53] | 1.13 [ 0.68, 1.50] | 0.50 | |  |
| Infarct-edema growth rate, mL/day | 8.16 [2.13, 20.01] | 28.41 [10.82, 45.99] | **0.003** | |  |
| CT scans performed closest to LKW (CT_baseline_) and closest to 48 hours from LKW (CT_endpoint_) were selected to calculate infarct-edema growth rate within 2 days of LKW. | | | |  |  |

| **Supplemental Table 2. Clinical and radiographic features between good and poor outcomes, in patients with at least 2 CT within 2 days of LKW** | | | | | |
| --- | --- | --- | --- | --- | --- |
| Characteristics | Good outcome  (n = 43) | Poor outcome  (n = 24) | *P* value | |  |
| Age, y | 60 [55, 72] | 58.5 [49, 72] | 0.33 | |  |
| Female | 19 (44) | 10 (42) | 1.00 | |  |
| African American | 13 (33) | 21 (52) | 0.17 | |  |
| Prior comorbidities | | | | | |
| Prior stroke | 8 (19) | 9 (38) | 0.14 | |  |
| Hypertension | 30 (70) | 20 (83) | 0.26 | |  |
| Coronary artery disease | 11 (26) | 9 (38) | 0.41 | |  |
| Atrial fibrillation/flutter | 7 (16) | 3 (13) | 1.00 | |  |
| Diabetes Mellitus | 14 (33) | 9 (38) | 0.79 | |  |
| Hyperlipidemia | 23 (53) | 10 (42) | 0.45 | |  |
| Imaging findings^a^ |  |  |  | |  |
| CT_baseline_ infarct-edema volume, mL​ | 6.6 [0, 21.2] | 2.2 [0, 10.1] | 0.10 | |  |
| CT_endpoint_ infarct-edema volume, mL | 23.0 [15.9, 32.4] | 35.5 [17.8, 44.6] | **0.047** | |  |
| Time to CT_baseline_, day | 0.37 [0.12, 1.01] | 0.29 [0.13, 0.96] | 0.82 | |  |
| Time to CT_endpoint_, day | 1.70 [1.28, 2.06] | 1.40 [0.94, 1.95] | 0.08 | |  |
| Δ Time, day | 1.2 [0.6, 1.6] | 0.8 [0.5, 1.3] | 0.11 | |  |
| Infarct-edema growth rate, mL/day | 6.0 [1.4, 16.5] | 24.6 [15.1, 46.6] | **< 0.0001** | |  |
| Infarct territory |  |  | **0.048** | |  |
| Bilateral or multiple | 26 (60) | 18 (75) | 0.29 | |  |
| PICA only | 14 (33) | 2 (8) | **0.04** | |  |
| AICA only | 0 (0) | 2 (8) | 0.13 | |  |
| SCA only | 3 (7) | 2 (8) | 1.00 | |  |
| Brainstem infarct | 10 (23) | 13 (54) | **0.02** | |  |
| Hemorrhagic transformation^b^ | 5 (12) | 4 (17) | 0.71 | |  |
| Hospital course | | | | | |
| Time to ED presentation | 0.37 [0.12, 1.01] | 0.29 [0.13, 0.96] | 0.83 | |  |
| tPA | 5 (12) | 2 (8) | 1.00 | |  |
| Mechanical thrombectomy | 3 (7) | 6 (25) | 0.06 | |  |
| GCS | 15 [14, 15] | 8.5 [5, 13.8] | **< 0.0001** | |  |
| NIHSS | 4 [2, 9] | 21.5 [12, 28] | **< 0.0001** | |  |
| Glucose, mg/dL | 154 [118, 221] | 170 [124, 229] | 0.72 | |  |
| Creatinine, mg/dL | 0.9 [0.8, 1.2] | 0.8 [0.7, 1.2] | 0.63 | |  |
| Surgical intervention, any | 12 (28) | 10 (42) | 0.29 | |  |
| Intervention type |  |  |  | |  |
| Osmotic | 19 (44) | 18 (75) | **0.02** | |  |
| EVD | 11 (26) | 9 (38) | 0.41 | |  |
| SDC | 10 (23) | 8 (33) | 0.40 | |  |
| Time to osmotic, day | 2.3 [1.5, 3.5] | 1.4 [1.1, 5.4] | 0.43 | |  |
| Time to EVD, day | 2.2 [1.5, 3.0] | 1.5 [1.1, 2.4] | 0.26 | |  |
| Time to SDC, day | 2.5 [1.8, 3.9] | 1.3 [1.1, 2.3] | **0.03** | |  |
| Clinical outcome |  |  |  | |  |
| Δ GCS | 0 [0, 1] | -1.5 [-4, 0.8] | **0.003** | |  |
| Δ NIHSS | -1 [-4, 0.5] | 13 [1, 17.8] | **< 0.0001** | |  |
| Data presented are number (%) for categorical and median [IQR] for continuous variables. PICA indicates posterior inferior cerebellar artery; AICA, anterior inferior cerebellar artery; SCA, superior cerebellar artery; tPA, tissue plasminogen activator; GCS, Glasgow Coma Scale; NIHSS, National Institutes of Health Stroke Scale.  ^a^ CT scans performed closest to LKW (CT_baseline_) and closest to 48 hours from LKW (CT_endpoint_) were selected to calculate infarct-edema growth rate within 2 days of LKW  ^b^ Asymptomatic, hemorrhagic infarction type 1 or hemorrhagic infarction type 2 based on the European Cooperative Acute Stroke Study II classification | | | |  |  |
